# Structural Interactions of β-Lactam Antibiotics with Mammalian Serum Albumins

**DOI:** 10.3390/ijms27020776

**Published:** 2026-01-13

**Authors:** Kajetan Duszynski, Bartosz Sekula, Julita Talaj, Anna Bujacz

**Affiliations:** 1 Institute of Molecular and Industrial Biotechnology, Lodz University of Technology, Stefanowskiego 2/22, 90-573 Lodz, Poland; 2 Department of Molecular Biology of Cancer, Medical University of Lodz, Mazowiecka 6/8, 92-215 Lodz, Poland

**Keywords:** β-lactam antibiotics transport, penicillin, drug binding, serum albumin, crystal structures

## Abstract

The Bactericidal action of β-lactam antibiotics is related to covalent modification of transpeptidases, enzymes that take part in the synthesis of bacterial cell wall. The β-lactam moiety mimics the transpeptidase substrate and irreversibly inhibits the enzyme. In penicillin and cephalosporin, the β-lactam ring is coupled with a five-membered thiazolidine ring or a six-membered dihydrothiazine ring, respectively. In the case of penicillins, such conjunction causes higher tension of this bicyclic moiety; therefore, the β-lactam ring can be hydrolyzed in certain conditions, inactivating the antibiotic. Serum albumin is known for its drug binding capabilities, which enable it to transport pharmaceuticals through the circulatory system. Penicillins and cephalosporins are no exception in this aspect, and they are also carried by serum albumin in the bloodstream. In this study, we structurally investigate the ability of three serum albumins—equine (ESA), caprine (CSA), and ovine (OSA)—to bind two penicillins, ampicillin (Amp) and oxacillin (Oxa), and two cephalosporins, cefaclor (Cef) and cephalosporin C (Csc). The crystal structures of these mammalian serum albumin complexes shed new light on the albumin binding properties of β-lactam antibiotics, showing one common binding site for Amp, Oxa, and Cef in Fatty Acid Site 6 (FA6), and a second cefaclor molecule bound in domain I of the equine serum albumin. It was surprising that these antibiotics are not bound in the main drug binding site. However, cephalosporin C is bound in OSA Drug Site 1 (DS1).

## 1. Introduction

β-lactam antibiotics are a class of antimicrobial agents with a broad spectrum of action and a wide therapeutic index [1]. They have gained popularity due to their high efficiency against bacterial infections and low therapy costs. They are applied in the treatment of various conditions, including skin, ear, and upper respiratory tract infections [2]. Historically, the first identified and isolated β-lactam antibiotic was penicillin [3], which in nature is synthesized by the fungi *Penicillium* sp. [4,5,6]. However, a milestone in antibiotic research that has led to the improvement of novel and highly effective semi-synthetic β-lactam antibiotics was the discovery of 6-aminopenicillanic acid and the development of its production methodology [7].

There are several major groups in the β-lactam antibiotic family [8]: (i) penicillins, with the five-membered thiazolidine ring; (ii) cephalosporins, with the six-membered dihydrothiazine ring; (iii) monobactams, in which the β-lactam ring is not coupled with any other cyclic moiety; (iv) carbapenems—structurally more similar to penicillin, but with one double bond in the five-membered ring and the sulfur atom substituted with carbon; and (v) β-lactamase inhibitors, with the biological activity more directed at a decrease of the bacterial resistance to β-lactam antibiotics action than at their direct bactericidal activity [9].

The high reactivity of penicillins is related to the lability of the amide bond of the β-lactam ring, which makes this class of compounds very susceptible to nucleophilic attack [10]. This lability is even more increased by the highly stretched and very reactive bicyclic moiety of the β-lactam ring coupled with the five-membered thiazolidine ring. It forces a non-planar conformation of the moiety with a large angle and torsional rotation of penicillins [11]. Fragility of the β-lactam ring is more characteristic for the penicillin group than, e.g., cephalosporins, where the cyclic moiety, built of the six-membered dihydrothiazine ring with one double bond, presents lower conformational stretch and thus higher stability [9].

The antimicrobial activity of penicillins and cephalosporins, as of other β-lactam antibiotics, is linked with the formation of covalent and stable bonds with transpeptidases, bacterial peptidoglycan-synthesizing proteins responsible for the formation of cross-linking D-alanine–D-alanine bonds in bacterial cell walls [12,13]. However, some bacterial strains have developed resistance mechanisms against β-lactam antibiotics action [14]: (i) production of β-lactam-degrading enzymes—β-lactamases; (ii) modification of the active site of transpeptidases, which prevents covalent linkage with antibiotics; (iii) alteration of membrane permeability or development of antibiotic efflux—both of which prevent undesired transpeptidase complexing. The medical application of penicillins is linked with the relatively high risk of side effects and adverse reactions [15]. Penicillin G is noted as the most common antibiotic that induces acute and sub-acute allergic reactions [2]. The new generation of penicillins has been developed to reduce immune response and enable oral dosing of the drug. Semisynthetic penicillins produced today, e.g., amoxicillin and ampicillin, broaden their antimicrobial range and have improved side effect profile, lower toxicity, and superior pharmacokinetics [16,17,18].

Cephalosporins are represented by a wide range of molecules, which vary according to three individual moieties. Different substituents directly affect the affinity of cephalosporin to albumins [19]. Cephalosporin C, one of the first characterized cephalosporins, was discovered by chance after being eluted from the column purifying cephalosporin N [20,21,22]. It has a more hydrophilic profile in comparison with Penicillin G due to its D-α-aminoadipic acid connected to the cephem scaffold. Cephalosporin C quickly became a prototype for future cephalosporins due to its activity against penicillin-resistant cultures [23]. However, the mechanism of β-lactam ring inhibition is preceded by elimination of the acetate group from the cephem scaffold. It evokes lower efficiency of antimicrobial activity of Cephalosporin C. Cefaclor belongs to the first group of the second generation of cephalosporins [8] and has been widely used in infections of the respiratory tract, urinary tract, and soft tissues [24]. Cephalosporins exhibit higher resistance to β-lactamases than penicillins, due to the more planar cephem scaffold conformation [22]. In an aqueous environment, acid-catalyzed hydrolysis of cephalosporins and penicillins is relatively slow. The degree of β-lactam antibiotics hydrolysis is highly dependent on pH and temperature; base-catalyzed hydrolysis is faster than hydrolysis in acidic and neutral pH [25].

Given the pharmacological significance of β-lactam antibiotics, understanding their interactions with serum albumin, an essential macromolecule in the mammalian body, is of particular interest. Although this group of antibiotics has been known over 80 years, structural insights into their interactions with serum albumin have been scarce.

Serum albumin, the major transport protein of vertebrate plasma, is a monomeric protein with three structurally similar helical domains (I–III), which form a heart-shaped molecule with a characteristic pattern of 17 disulfide bridges [26]. Each albumin domain is formed by two homologous subdomains (A and B). Two big pockets, called Drug Site 1 and 2 (DS), which can bind a number of small molecules, have been identified in subdomain IIA and IIIA of albumin [27,28,29]. Other major binding pockets of albumin are very often referred to as fatty acid sites (FAs) [30,31,32]. These binding pockets are responsible for transport in the circulatory system of not only fatty acids but also drug molecules and hormones [33,34]. In addition to human serum albumin (HSA), other mammalian serum albumins in complexes with drugs have been studied for more than 10 years [35,36,37]. As one of the most abundant plasma proteins with highly developed binding properties and capacity, serum albumin is thought to be a key factor in the regulation of drug distribution and excretion processes [38].

Structural data showing antibiotic binding to serum albumin is limited to the crystal structure of HSA in complex with fusidic acid [39]. To date, there is only one publication describing crystal structures of HSA complexes with two β-lactam antibiotics: cefazolin and ceftriaxone (Cefl, Ceft) [40]. They are bound in the FA1 pocket of subdomain IB, the same as the site of fusidic acid binding. This binding site of HSA interacts also with bilirubin and heme [39,41]. However, docking and displacement studies designate DS1 to be the binding site for compounds with β-lactam moiety [42,43]. Our structural studies of mammalian serum albumins (equine (ESA), caprine (CSA), leporine (LSA), and ovine (OSA)) reveal a large diversity in drug binding sites depending on species [44,45,46]. 

**Figure 1:**
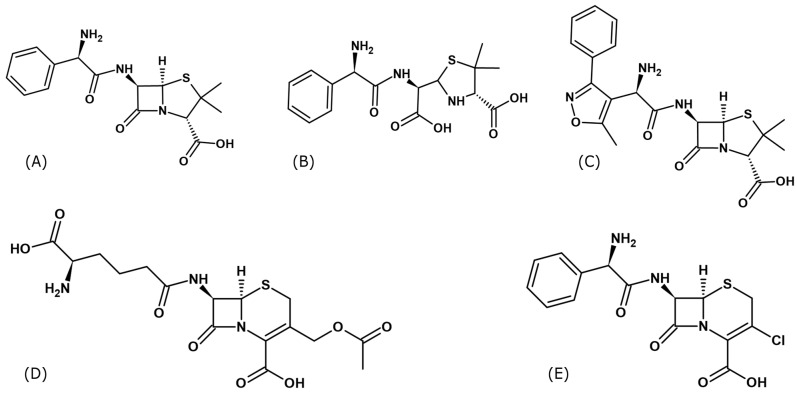
Structural formulas of β-lactams antibiotics used to form complexes of serum albumins: (**A**) Ampicillin (Amp), (**B**) Hydrolyzed form of ampicillin (Amp_h_), (**C**) Oxacillin (Oxa), (**D**) Cephalosporin C (Csc) and (**E**) Cefaclor (Cef) (Formulas were drawn using ChemSketch v.2024.2.3.).

A novelty presented in this manuscript is determination, for the first time, of the spatial structures of several serum albumins complexes with ampicillin (Amp) [47,48], oxacillin (Oxa) [49,50,51], cephalosporin C (Csc) [43], and cefaclor (Cef) [52,53] (Figure 1). 

In this work, we describe eight crystal structures of serum albumin from various animals: horse (ESA), sheep (OSA) and goat (CSA) complexed with Amp, Oxa, Csc and Cef, revealing the precise binding sites of serum albumin interacting with these β-lactam antibiotics. The results we obtained will contribute to a better understanding of the β-lactam antibiotics distribution, highlighting that these hydrolysis-sensitive drugs may degrade into inactive compounds circulating in the body rather than remaining as the therapeutically active molecules. Therefore, we also discuss the cause of finding a hydrolyzed form of ampicillin in some of the obtained structures.

## 2. Results

### 2.1. Penicillins Bound in Domain II of Albumins at Fatty Acid Site 6 (FA6)

**Figure 2:**
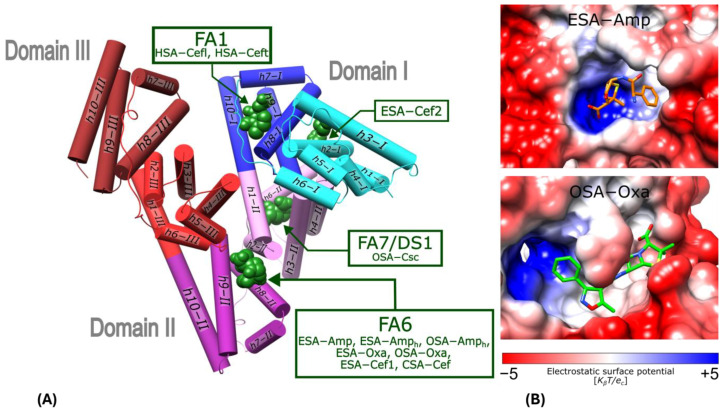
Binding sites for β-lactam antibiotics in serum albumins: human (HSA; Cefl and Ceft, PDB ID: 8XYA and 8XYB) [40], equine (ESA; Amp, Amp_h_, Oxa and Cef), ovine (OSA; Amp_h_ and Oxa), and caprine (CSA; Cef and Csc). (**A**) Serum albumin structural topology; helices are presented as cylinders, with each serum albumin subdomain colored as follows: IA (cyan), IB (blue), IIA (pink), IIB (violet), IIIA (red), and IIIB (brown). (**B**) Surface electrostatic potential of ESA and OSA FA6 pocket with bound Amp or Oxa.

The crystal structures of ESA and OSA with Amp and Oxa present a single, common binding location of both bactericidal agents. The binding cleft responsible for the interactions with these antibiotics is located at the interface of subdomain IIA and IIB (Figure 2) and is called FA6 [30]. This site was recognized in bovine (BSA), equine (ESA), and leporine (LSA) serum albumins as the lower affinity binding location for naproxen (Nps) [35,36], as well as for ibuprofen (Ibu) in HSA and ESA [29,37]. Mainly saturated and unsaturated medium- and long-chain fatty acids have an affinity for this pocket of HSA [30,31,32]. A phosphorodithioate analogue of cyclic phosphatidic acid (Myr-2S-cPA) was found to be bound in the analogous FA6 pocket of ESA [54].

The FA6 binding pocket has the shape of a niche, with the bottom wall built of residues from the h8-II and h9-II of subdomain IIB (Figure 2A). It is occluded by residues Arg208 and Asp323, which form a kind of buckle between the subdomains IIA and IIB, best visible in ESA. This pocket covers a rather hydrophobic central part and can be divided into two side compartments. In the left-hand-side compartment, the *N*-terminal part of h6-III creates the positively charged patch, which is especially ideal for the interactions with anionic ligands in ESA (Figure 2B upper panel), but in OSA, the whole FA6 pocket is more hydrophobic, allowing for different conformations of the bound ligands (Figure 2B lower panel).

Residues from h9-II also contribute to the shape of the left-hand side of FA6. The upper part of the central compartment of FA6 is walled by residues from h1-II and h2-II of subdomain IIA, while the bottom is built by h8-II. The right-hand side compartment of FA6 is constituted by h3-II. For binding the shorter fatty acids, this compartment is referred to as FA6 [30].

**Figure 3:**
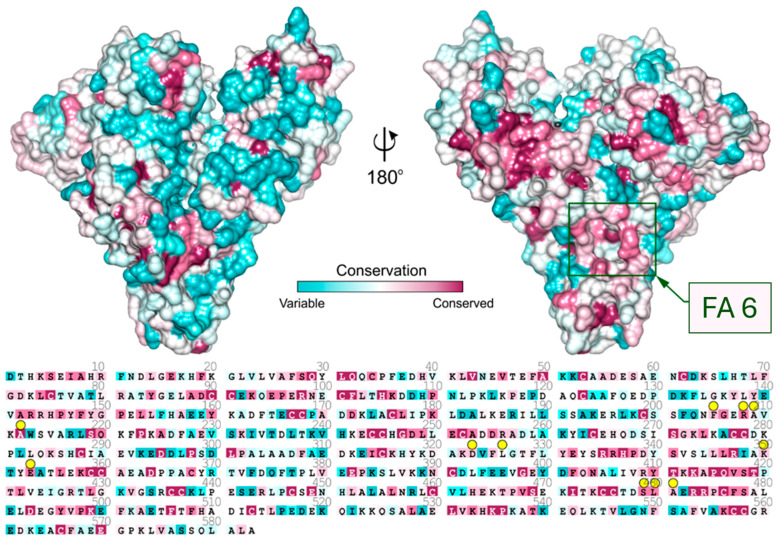
Sequence conservation of serum albumin mapped on the surface of the ESA structure. Residues interacting with Amp, Oxa, and Cef are marked with yellow circles. The location of the FA6 site is marked with a green rectangle.

Analysis of sequence conservation of known mammalian serum albumins (Figure 3) shows that the entrance to the FA6 pocket is built of highly conserved amino acids. The residues that are placed in the left-hand side compartment exhibit much higher conservation than residues placed on the other side of FA6. Charged residues at the entrance to this pocket are responsible for ligand recognition, and they are almost identical in all investigated albumins. Crystal structures show that either ampicillin, oxacillin, or cefaclor is bound in the FA6 pocket of equine, caprine, and ovine serum albumins (Figure 2).

### 2.2. Ampicillin Binding Mode

**Figure 4:**
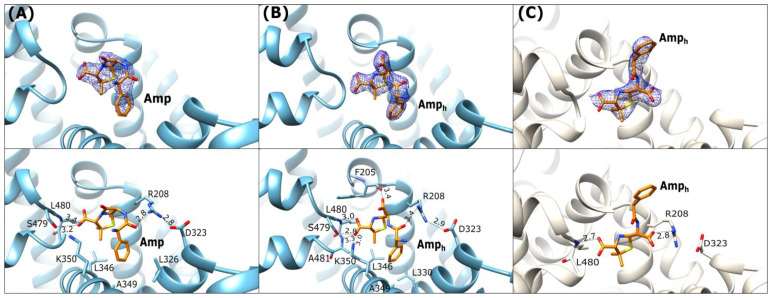
Ampicillin binding mode: (**A**) ESA-Amp (blue), (**B**) ESA-Amp_h_ (blue), (**C**) OSA-Amp_h_ (light beige). **Upper panels**: *2Fo-Fc* electron density maps contoured at 1 σ (blue mesh). The **lower panels** show detailed interactions in [Å] of the bound ligands within the binding pocket FA6.

Three crystal structures of albumins in complex with ampicillin (Amp) were determined in the course of the present work. We determined two structures of ESA (ESA-Amp and ESA-Amp_h_), as well as a single structure of OSA (OSA-Amp_h_). These complexes show two forms of the bound ligands, designated Amp and Amp_h_, with either an intact or opened β-lactam ring, respectively (Figure 1A,B). The ESA-Amp complex obtained by short soaking presents a bound antibiotic with the preserved β-lactam ring (Figure 4A).

In all three structures the position of the five-membered thiazolidine ring is almost identical, with the carboxyl group of Amp interacting with the *N*-terminal part of h6-III and the two methyl substituents of the ring oriented towards the hydrophobic concave surface of the pocket.

In ESA complexes, the carboxylic group on the thiazolidine ring of Amp and Amp_h_ creates hydrogen bonds with the main chain peptide nitrogen of Leu480 and the side chain hydroxyl group of Ser479 (Figure 4A,B). In ESA-Amp_h_ the same carboxyl group additionally interacts with backbone amide of Ala481 and Nζ of Lys350. The phenyl ring of Amp_h_ in the OSA complex is oriented outside of FA6 (Figure 4C) and is not involved in any significant interactions with the protein. In ESA complexes, this site is occupied by ions from the crystallization or cryoprotection solutions malonate and malate in ESA-Amp and ESA-Amp_h_, respectively. In the OSA-Amp_h_ complex, the carboxylic group of the hydrolyzed β-lactam ring is H-bonded with Arg208 (Figure 4C). The equivalent arginine in the ESA-Amp and ESA-Amp_h_ structures interacts with the carbonyl oxygen of the amide Amp group. The salt bridge closing the entrance to the FA6 pocket, observed in ESA-Amp and ESA-Amp_h_ between Arg208 and Asp323 (Figure 4A), is looser in the OSA-Amp_h_ structure, due to the interaction of Arg208 with the carboxylic group of the hydrolyzed β-lactam ring.

In ESA-Amp, Amp adopts a highly bent, horseshoe-like conformation, with the phenyl ring placed in the hydrophobic environment close to h8-II. In ESA-Amp_h_, the ligand is also bent, and its carboxylic group of the hydrolyzed β-lactam ring creates a hydrogen bond with the carbonyl oxygen of the main chain Phe205. Additionally, the carboxyl group of the hydrolyzed β-lactam moiety interacts with a malonic ion, which gets into the crystal from the cryoprotectant solution. In the OSA-Amp_h_ complex, Amp_h_ adopts a more extended, almost linear conformation. A comparison of Amp and Amp_h_ interactions with ESA and OSA shows that the opening of the strained β-lactam ring influences the changes in the protein–ligand interactions.

### 2.3. Oxacillin Binding Mode

**Figure 5:**
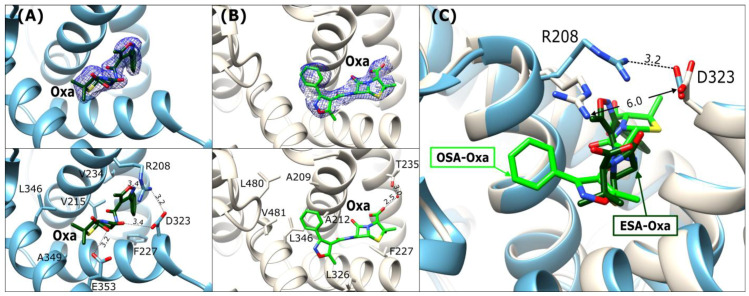
Oxacillin binding mode in FA6: (**A**) ESA-Oxa (blue) and (**B**) OSA-Oxa (light beige). Upper panels: *2Fo-Fc* electron density maps (blue mesh contoured at 1 σ). The lower panels show detailed interactions in [Å] of bound antibiotics within the FA6 binding sites. (**C**) Superposition of Oxa in ESA and OSA; the distances in [Å] between R208 and D323 are marked.

In the structures of ESA-Oxa and OSA-Oxa (Figure 5A,B), the antibiotic is placed in the FA6 binding cleft, which is the same as the binding site for ampicillin. The intact β-lactam ring of the bound drug is embedded deeply into the binding cleft. The Oxa molecules in ESA and OSA are located in the same place; however, they have a different orientation in both complexes and adopt a more linear structure in comparison to the bent Amp molecule in the ESA-Amp and OSA-Amp_h_ complexes (Figure 5).

The ESA-Oxa isoxazole ring is located near the Arg208-Asp323 salt bridge, with its oxygen forming a hydrogen bond with the guanidine moiety nitrogen of Arg208. On the other part of the oxacillin molecule, the carboxyl group of the thiazolidine ring interacts with the carboxyl group of Glu353 (Figure 5A). The phenyl substituent on the isoxazole ring is embedded in the vicinity of the hydrophobic amino acid side chains in this pocket. Two methyl groups adjacent to the thiazolidine ring are oriented toward nonpolar amino acids.

In the OSA-Oxa complex, the ligand molecule is rotated ~180^0^ around its amide group in comparison to Oxa in ESA-Oxa (Figure 5A,B). The Arg208-Asp323 salt bridge visible in the ESA structure is disrupted in the OSA complex (Figure 5C), probably because of the presence of a malonate ion in this binding pocket, resulting in a more open cleft. The phenyl substituent of Oxa is positioned within the hydrophobic region of the pocket, and the carboxyl group of the thiazolidine ring establishes a water-mediated hydrogen bond with the side-chain oxygen of Thr235. Despite the different orientations of Oxa in these two structures, its nonpolar regions (i.e., the phenyl of the isoxazole moiety and the methyl groups) consistently face the hydrophobic part of the pocket.

### 2.4. Cefaclor (Cef) Binding Mode

**Figure 6:**
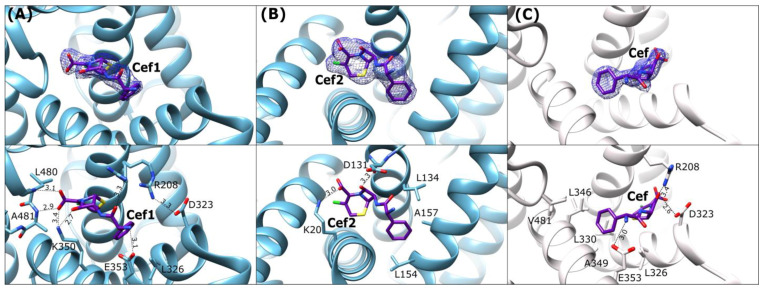
Cefaclor binding mode: (**A**) ESA-Cef1 (blue), (**B**) ESA-Cef2 (blue), and (**C**) CSA-Cef (light gray). **Upper panels**: *2Fo-Fc* electron density maps (blue mesh contoured at 1 σ). **Lower panels** show detailed interactions in [Å] of the bound antibiotic within the binding sites.

Two interaction sites between the albumin and cefaclor (Cef) molecules were recognized in the ESA–Cef structure (Figure 6A,B). Both Cef molecules are bound in their intact, non-hydrolyzed form. The first cefaclor (Cef1) molecule (Figure 6A) occupies the pocket corresponding to the FA6 cavity, similar to Oxa and Amp. In this binding site, the ligand adopts a horseshoe-like conformation positioned closer to the IIA and IIIB subdomains. The nitrogen atom Nζ of Lys350 creates a hydrogen bond with the carbonyl oxygen of the β—lactam ring and participates also in hydrogen bonding with an oxygen atom from the carboxyl group attached to the Cef1 dihydrothiazine ring. The same carboxyl group creates hydrogen bonds with the nitrogen atoms of the main chain peptide bonds of Leu480 and Ala481.

All these interactions hold the ligand in this position. The amine group and the carbonyl oxygen of an amide bond from the remaining part of the Cef1 molecule interact with the carboxyl group of Glu353 and the guanidine moiety of Arg208, respectively. The other conformer of Arg208 is involved in salt bridge interactions with Asp323. The Cef1 molecule adopts in this pocket a horseshoe-like conformation, similar to ampicillin, and the phenyl moiety of Cef1 is directed toward the nonpolar region of the FA6 cavity.

The second cefaclor molecule (Cef2) is bound between subdomains IA and IB in the cavity composed of helices h2-I, h3-I, h8-I, and h9-I. In caprine serum albumin, 3,5-diiodosalicylic acid (Dis) and diclofenac (Dic) were bound in an equivalent location [45,46]. The Cef2 molecule is completely embedded into this binding pocket and is not as much bent as Cef1 in FA6. The observed H–bond is created between the amino group of Lys20 and the carboxyl group of Cef2, and the second weak contact is observed between Cef2 amide oxygen and the carboxyl group of Asp131. The phenyl ring of the ligand is surrounded by the side chains of hydrophobic residues (Figure 6B).

In the CSA-Cef structure, a single cefaclor molecule is bound in its intact form, adopting a horseshoe-like conformation (Figure 6C). The molecule is placed in the FA6 site, between the helices h2-II, h8-II, and h9-II, similarly to the binding observed in ESA-Cef. Despite sharing a common site, Cef is rotated by ~180° in the CSA-Cef complex, with a ~5 Å translational shift relative to Cef1 in the ESA-Cef complex. Additionally, the pocket in CSA-Cef is partially occupied by a citrate ion from the crystallization buffer, which probably caused a shift of the ligand in this large cavity. The phenyl group takes up a hydrophobic part of the pocket that includes residues L326, L330, L346, L481, and A349. Several hydrogen bonds stabilize Cef at this site: the primary amine forms a hydrogen bond with the carboxylic group of Glu353. The carbonyl oxygen of the β-lactam ring engages in hydrogen bonding with the side chains of Arg208 and Asp323, thereby disrupting the salt bridge between them (Figure 6C).

### 2.5. Cephalosporin C (Csc) Binding Mode

**Figure 7:**
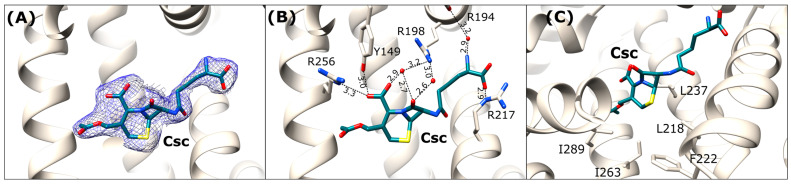
Cephalosporin C binding site in OSA-Csc (light beige). (**A**) Csc ligand in *2Fo-Fc* electron density map (blue mesh contoured at 1 σ); (**B**) Detailed interactions in [Å] of the bound antibiotic within the DS1 binding site; (**C**) Hydrophobic amino acids surrounding the dihydrothiazine ring of Csc in the binding pocket.

Cephalosporin C (Csc) in the OSA-Csc complex has a single binding site localized in DS1/FA7 (Figure 7), unlike the other investigated β-lactam antibiotics. Compared with Cef, Csc displays a more aliphatic character, owing to its non-aromatic D-α-aminoadipic acid side chain and therefore adopts an extended, linear conformation. The contacts stabilizing this extended chain involve interactions formed between its primary amine with the peptide oxygen of Arg194 via a water molecule, as well as hydrogen bonding between its carboxyl group and the guanidine moiety of Arg217 (Figure 7).

In contrast to the rather flexible D-α-aminoadipic acid moiety, the six-membered dihydrothiazine ring is tightly bound within the binding pocket. The hydrophobic part of the ring is oriented towards the side chains of non-polar amino acids, including Leu218, Phe222, Ile237, Ile263, and Ile289 (Figure 7C). The carboxyl group of the dihydrothiazine ring forms hydrogen bonds with the hydroxyl group of Tyr149, the guanidine moiety of Arg256, and a water-mediated interaction with the side chain of Arg198. Additionally, the carbonyl oxygen of β-lactam ring interacts via two water molecules with the guanidine group of Arg198 (Figure 7B).

## 3. Discussion

### 3.1. Comparison of the β-Lactam Antibiotics Binding Sites in Serum Albumins

Both penicillins (Amp and Oxa) are bound by equine (ESA) and ovine (OSA) albumin in the same FA6 cleft, with either an intact or hydrolyzed β-lactam moiety. Structurally, Oxa differs from Amp by the presence of a rigid 5-methylisoxazole ring (Figure 1). Ampicillin has only an amine group neighboring the phenyl ring and thus has more conformational flexibility in this part of the molecule than Oxa. In the case of ESA complexes, both molecules, Amp and Amp_h_, pose a similar bent conformation. In the case of OSA, Amp_h_ rotates its phenyl ring outside of the binding cleft, and the ligand adopts an extended conformation. The flat isoxazole ring of Oxa introduces rigidity to the molecule, forcing a more extended conformation. Conformational differences of amino acid side chains in the FA6 pocket, as well as the flexibility or rigidity of the ligands and the presence of some components of the crystallization buffer, can influence different antibiotic conformations. The Oxa molecules are located in the same compartment of the FA6 pocket, but in ESA and OSA, the ligand adopts a reversed orientation. The entrance to the FA6 pocket is modulated by an interaction between the side chains of Arg208 and Asp323. This Arg208–Asp323 salt bridge is maintained in all ESA presented complexes but disrupted in OSA and CSA in complexes with Amp_h_ and Cef. Its presence probably depends on the relative arrangement of subdomains or molecules in the crystal lattice. Additionally, other factors cannot be excluded, such as ligand size and properties, crystallization conditions, and the solvent environment.

Cefaclor (Cef) and cephalosporin C (Csc) are less prone to hydrolysis of their β-lactam moiety due to the more stable six-membered dihydrothiazine ring instead of a five-membered thiazolidine ring. Unlike in the β-lactam antibiotics, such as Amp, Oxa and Cef, which are bound in the FA6 pocket, cephalosporin C in the OSA-Csc complex is bound in the DS1 pocket, and in the FA6, the myristic acid was found.

Recently, two crystal structures of HSA complexes with cephalosporins, cefazolin (PDB ID: 8YXA) and ceftriaxone (PDB ID: 8YXB), in the presence of myristic acid were published [40]. Both antibiotics are bound in the FA1 pocket, but myristic acid is bound in the FA6 cavity, similar to Myr in the OSA-Csc complex. In physiological or different crystallization conditions, the presence of the other ligands, e.g., fatty acids, can cause β-lactam antibiotics to bind to other pockets.

### 3.2. Hydrolysis of Penicillins β-Lactam Ring

We attempted to find an answer for why, in some structures, we observe penicillin bound in a form with an intact β-lactam ring, but in some others with a hydrolyzed β-lactam ring. In the case of penicillin bound inside the FA6 site, the ligand is stabilized by a network of hydrogen bonds of the carboxyl group coupled with the thiazolidine ring, and the placement of the phenyl ring in the hydrophobic milieu hypothetically exposes the β-lactam ring for the nucleophilic attack [55,56]. The residue in FA6 that could match this requirement is Ser479, which is placed in the vicinity of the thiazolidine ring, but it is too far for the interactions with the β-lactam ring and therefore, excluding its role as a potential catalytic residue. The β-lactam ring in cefaclor (Cef) is closer to the hydroxyl group of Ser479 in comparison to Amp and Oxa and remains unimpaired. Therefore, hydrolysis might most likely occur only through the non-covalent mechanism before complex creation under the influence of favorable components and conditions of the crystallization solution. Since there is no potential residue that is able to perform the nucleophilic attack on the cyclic moiety of Amp, a water molecule or some agent from the crystallization solution might perform the attack before or during co-crystallization. Our structural findings show that albumin is unable to actively participate in the hydrolysis of the β-lactam ring.

We postulate that the main factor responsible for the presence of hydrolyzed β-lactam ring in two structures of albumin complexes with penicillins is long incubation and the presence of high ionic strength components of the crystallization buffer. Therefore, the antibiotic administration route has a significant impact on therapeutic effectiveness. It was reported that the oral absorption dose is 33% for oxacillin and 33–54% for ampicillin [57]. Additionally, mainly low pH present in the oral administration route is a potential factor of ß-lactam ring disruption [25]. The β-lactam ring of penicillin is susceptible not only to hydrolysis but also to a transamination reaction with the ζ-amine groups of lysines on the albumin surface to create unwanted adducts in some patients treated by very high doses of penicillin. These adducts are the main antigenic determinants of allergy to antibiotics [58].

## 4. Materials and Methods

### 4.1. Complex Formation and Crystallization

Commercially available ESA (Equitech-Bio Inc., Kerrville, TX, USA), OSA, and CSA (Sigma-Aldrich, St Louis, MO, USA) were also purified in order to remove fatty acids and the dimer fraction. Briefly, the proteins were treated with activated charcoal and subjected to size exclusion chromatography with a Superdex 200 prep grade column XK 16/100 connected to the AKTA FPLC system (Amersham Biosciences, Uppsala, Sweden) in the running buffer (100 mM NaCl, 10 mM Tris at pH 7.4). Proteins were concentrated on the VivaSpin microconcentrators’ 10 kDa cutoff (Sartorius, Goettingen, Germany) to the final concentration of 1.2 mM of ESA and 0.8 mM of OSA and CSA.

The first albumin complex was obtained in this study by overnight incubation of ESA solution with ampicillin in the protein-to-antibiotic molar ratio 1:10 at room temperature. This complex was crystallized in the presence of 80% *v*/*v* Tacsimate pH 6.0. After solving the crystal structure, we found in electron density maps a hydrolyzed form of ampicillin (ESA-Amp_h_). We concluded that the long contact of β-lactam antibiotic with the ionic crystallization mother liquor caused hydrolysis of the β-lactam ring; thus, we decided to use a soaking procedure for the other complexes. ESA-Amp, ESA-Oxa, ESA-Cef, and CSA-Cef were obtained by soaking native albumin crystals in crystallization solutions with β-lactam antibiotics in a molar stoichiometry 1:10, incubating each complex for 1.5–3 h before diffraction data collection. We have established a stoichiometric ratio of 1:10 based on many previously created albumin-drug complexes to ensure full ligand occupancy of the all potential serum al-bumins binding sites. 

The albumins were crystallized by the hanging drop diffusion method at 20 °C. Crystals with the best morphology were obtained in the following conditions: ESA—2.0 M ammonium sulfate and 0.1 M acetate buffer at pH 5.0, OSA—grown in 80% Tacsimate at pH 7.25 and 2% of PEG 400, CSA crystals were obtained in the presence of 30% Jeffamine pH 7.0, 0.1 M sodium citrate pH 5.0, and 0.1 M barium chloride. ESA crystals, grown in the conditions containing ammonium sulfate, were cryoprotected before the diffraction experiment by 85% Tacsimate at pH 6.0. Crystals of CSA and OSA did not require additional cryoprotection [59].

### 4.2. Diffraction Data Collection and Structure Refinement

Diffraction data were collected on beam lines BL14.1 and BL.14.2, BESSY [60] (Berlin, Germany), and PX14 in PETRA (Hamburg, Germany). Data were processed in XDS [61] to the final resolution of 2.50 Å (ESA-Amp), 2.32 Å (ESA-Amp_h_), 2.03 Å (ESA-Oxa), 2.12 Å (ESA-Cef), 1.76 Å (CSA-Cef), 2.6 Å (OSA-Amp_h_), 2.3 Å (OSA-Oxa) and 2.2 Å (OSA-Csc).

Structures were determined by molecular replacement using the program MOLREP [62], followed, in most cases, by rigid body refinement with REFMAC (v. 5.8.0430) [63,64] from the CCP4 (v. 9.0.012) package [65]. The starting models were structures previously determined by us: ESA-Dic (PDB ID: 4ZBQ) [36], OSA (PDB ID: 4LUF), and CSA (PDB ID: 5ORI) [46]. These starting models were prepared as follows: ligands and solvent molecules were deleted, and the atomic displacement parameters were set to 20 Å^2^. The ligands were fitted into the electron density maps in COOT (v. 0.9.8.95) [66] and subjected to several refinement cycles in REFMAC [63], PHENIX [67], followed by inspection and manual adjustment in COOT. At the later stages of refinement, TLS parameters [68,69] were introduced into the structures. 

Coordinates, restraints, and geometric libraries for Amp, Amp_h_, Oxa, Cef, and Csc were acquired from the PDB [70] or prepared in the SKETCHER program from the CCP4 package [65]. In the case of Cef, due to the lack of proper chirality centers in the ligand taken from PDB, the geometry restraints were established in the new file mon_lib.cif in the SKETCHER program. All ligands were refined against standard CCP4 libraries. The quality of refined structures was controlled by *R_work_*, *R_free_* [71] and geometric parameters. PROCHECK [72] and MolProbity [73] were used for the evaluation of the final models. A summary of the data collection and refinement statistics is given in Table 1.

### 4.3. Other Software Used

Molecular illustrations were created with PyMOL v. 2.3 [74] and UCSF Chimera (v. 1.17.3) [75]. Ramachandran plot was calculated in Rampage [76]. Sequence conservation was calculated in ConSurf [77] based on the sequence alignment performed in MUSCLE [78] under the MEGA7 [79], with sequences classified as belonging to the serum albumin family by InterPro [80]. Surface electrostatic potential was calculated in PDB2PQR (v. 3.6.1) and APBS (v. 3.4.1) [81,82].

## 5. Conclusions

Although serum albumin is one of the most extensively studied proteins, there was a lack of structural data focused on the binding of β-lactam antibiotics by albumins of other species than humans. The determined crystal structures of equine (ESA), caprine (CSA), and ovine (OSA) albumins in complexes with four β-lactam antibiotics—ampicillin (Amp), oxacillin (Oxa), cefaclor (Cef), and cephalosporin C (Csc)—give structural insights into the transport of this group of compounds and contribute to the understanding of the distribution mechanism of this protein in the body. Under the studied conditions, we observe specific common binding sites of two penicillin derivatives, Amp and Oxa, into the same pocket of ESA and OSA, a cleft placed between subdomain IIA and IIB called FA6. The same binding site in CSA and ESA was occupied by cefaclor, but in ESA, in addition to binding in the FA6 pocket, Cef also binds to a non-conventional site localized between the IA and IB subdomains. Only OSA showed another place for binding Csc—DS1, which, according to the literature, is the place to which most of the drugs have affinity. None of the bound β-lactam antibiotics in ESA, OSA, and CSA was localized where cefazolin and ceftriaxone are placed in HSA [40].

Serum albumin is among the proteins exhibiting the largest variations in their sequences, but the character of some binding sites is very similar, at least to some extent. The fatty acid site (FA6) is selective for binding this group of antibiotics, probably because all three albumins possess high similarity of amino acid residues in this long cleft, to which physiologically long fatty acids have affinity. This FA6 pocket also binds a number of profens: naproxen in BSA, ESA, and LSA [35], and ibuprofen in ESA [37] and in HSA [29].

The presented crystal structures not only reveal detailed binding sites of β-lactam antibiotics in complexes with ESA, OSA, and CSA, but also show no evidence of enzymatic activity of serum albumins. Our structural analyses indicate that some β-lactam antibiotics are sensitive to hydrolysis and should be administered under conditions that would prevent or at least minimize their unwanted hydrolysis.

## Figures and Tables

**Table 1:** X-ray data collection and structure refinement statistics.

Structure	ESA-Amp	ESA-Amp_h_	OSA-Amp_h_	ESA-Oxa	OSA-Oxa	ESA-Cef	CSA-Cef	OSA-Csc
**Data collection**								
Beamline	BESSY BL.14.1	BESSY BL.14.1	Petra PX14	BESSY BL.14.2	BESSY BL.14.2	BESSY BL.14.1	BESSY BL.14.1	BESSY BL.14.1
Wavelength (Å)	0.918	0.918	0.976	0.918	0.918	0.918	0.918	0.918
Temperature (K)	100	100	100	100	100	100	100	100
Space group	*P*6_1_	*P*6_1_	*P*3_2_21	*P*6_1_	*P*3_2_21	*P*6_1_	*P*2_1_2_1_2_1_	*P*3_2_21
Unit cell parameters *a*, *b*, *c* (Å), α, β, γ (°)	95.0, 95.1, 143.1 α = β = 90 γ = 120	93.5, 93.5, 141.7 α = β = 90 γ = 120	121.2, 121.2, 122.0 α = β = 90 γ = 120	93.7, 93.7, 141.2 α = β = 90 γ = 120	121.5, 121.5, 122.2 α = β = 90 γ = 120	94.8, 94.8, 142.6 α = β = 90 γ = 120	42.9, 67.6, 214.8 α = β = γ = 90	120.3, 120.3, 123.0 α = β = 90 γ = 120
Oscillation range (°)	0.5	0.5	0.1	0.2	0.1	0.2	0.1	0.1
Resolution (Å)	47.53–2.50 (2.60–2.50)	44.38–2.32 (2.46–2.32)	50.0–2.55 (2.71–2.55)	46.86–2.03 (2.13–2.03)	6.83–2.30 (2.44–2.30)	47.38–2.12 (2.25–2.12)	42.04–1.76 (1.88–1.76)	48.0–2.20 (2.33–2.20)
Reflections collected/unique	145,438/ 25,271	156,038/ 30,368	208,920/ 32,992	251,403/ 45,127	305,813/ 46,557	567,503/ 41,089	194,029/ 60,997	347,774/ 52,635
Completeness (%)	99.9 (100)	99.8 (99.3)	96.7 (80.7)	99.9 (99.6)	99.5(98.9)	99.8 (99.0)	98.0 (95.3)	99.8 (99.2)
*R*_merge_ (%)	7.8 (103.0)	8.8 (103.0)	8.1 (107.4)	5.8 (76.1)	15.8 (81.4)	9.9 (188.9)	4.1 (58.9)	10.4 (86.2)
<*I*/σ(*I)*>	18.9 (2.2)	15.3 (1.8)	15.0 (1.5)	21.8 (2.1)	13.2 (2.3)	22.7 (1.5)	19.7 (2.0)	14.5 (2.3)
**Refinement**								
*R_free_* reflections	1044	1520	1320	1129	1164	2052	2101	1316
No. of atoms (non-H)								
protein	4573	4577	4646	4587	4664	4598	4816	4695
ligands	62	58	91	158	97	121	50	141
solvent	101	137	67	219	247	139	571	492
*R*_work_/*R*_free_ (%)	20.1/26.3	17.4/24.5	16.5/22.3	16.1/20.6	16.8/21.7	18.7/24.9	18.0/22.8	15.8/20.9
Mean ADP (Å^2^)	83.5	56.9	81.6	48.4	46.0	57.2	41.2	60.7
RMSD from ideal geometry								
bond lengths (Å)	0.016	0.019	0.012	0.015	0.021	0.019	0.019	0.011
bond angles (^o^)	1.5	2.0	1.3	1.9	2.1	2.0	2.0	1.5
Ramachandran statistics (%)								
favored	96	96	97	98	99	96	97	98
allowed	4	4	3	2	1	4	3	2
outliers	0	0	0	0	0	0	0	0
PDB code	9S73	9S42	9SDJ	9S43	9SC5	7Q4X	9Q8U	9QCH

ADP = atomic displacement parameter. Values in parentheses refer to the highest resolution shell. R_merge_= ΣhΣj|Ihj − ⟨Ih⟩|/ΣhΣjIhj, where Ihj is the intensity of observation j of reflection h. R = Σh||Fo| − |Fc||/Σh|Fo| for all reflections, where *F_o_* and *F_c_* are observed and calculated structure factors, respectively. R_free_ is calculated analogously for the test reflections and is randomly selected and excluded from the refinement.

## Data Availability

The original contributions presented in this study are included in the article. Further inquiries can be directed to the corresponding author.

## Abbreviations

The following abbreviations are used in this manuscript:

Amp	ampicillin, (2S,5R,6R)-6-[[(2R)-2-amino-2-phenylacetyl]amino]-3,3-dimethyl-7-oxo-4-thia-1-azabicyclo [3.2.0]heptane-2-carboxylic acid
Amp_h_	hydrolyzed ampicillin, (2R,4S)-2-[(R)-[[(2-amino-2-phenylacetyl]amino-carboxymethyl]-5,5-dimethyl-1,3-thiazolidine-4-carboxylic acid
BSA	bovine serum albumin
Cef	cefaclor, (6R,7R)-7-{[(2R)-2-amino-2-phenylacetyl]amino}- 3-chloro-8-oxo-5-thia-1-azabicyclo [4.2.0]oct-2-ene-2-carboxylic acid
Cefl	cefazolin, (6*R*,7*R*)-3-{[(5-methyl-1,3,4-thiadiazol-2-yl)thio]methyl}-8-oxo-7-[(1*H*-tetrazol-1-ylacetyl) amino]-5-thia-1-azabicyclo[4.2.0]oct-2-ene-2-carboxylic acid
Ceft	ceftriaxone, (6*R*,7*R*)-7-{[(2*Z*)-2-(2-amino-1,3-thiazol-4-yl)->2-(methoxyimino) acetyl]amino}-3-{[(2-methyl-5,6-dioxo-1,2,5,6-tetra-hydro-1,2,4-triazin-3-yl)thio]methyl}-8-oxo-5-thia-1-azabicyclo[4.2.0]oct-2-ene-2-carboxylic acid
Csc	cephalosporin C, (6R,7R)-3-(acetyloxymethyl)-7-[[(5S)-5-amino-5-carboxypentanoyl]amino]-8-oxo-5-thia-1-azabicyclo[4.2.0]oct-2-ene-2-carboxylic acid
Dic	diclofenac, 2-[2-(2,6-dichloroanilino) phenyl]acetic acid
Dis	3,5-diiodosalicylic acid
CSA	caprine serum albumin
DS	drug site
ESA	equine serum albumin
FA	fatty acid
HSA	human serum albumin
LSA	leporine serum albumin
Nps	naproxen, (*S*)-2-(6-methoxynaphthalen-2-yl)propanoic acid
NSAID	nonsteroidal anti-inflammatory drug
OSA	ovine serum albumin
Oxa	oxacillin, (2S,5R,6R)-3,3-dimethyl-6-[(5-methyl-3-phenyl-1,2-oxazole-4-carbonyl) amino]-7-oxo-4-thia-1-azabi-cyclo [3.2.0]heptane -2-carboxylic acid.
